# A Review of Biotechnological Artemisinin Production in Plants

**DOI:** 10.3389/fpls.2017.01966

**Published:** 2017-11-15

**Authors:** Nur K. B. K. Ikram, Henrik T. Simonsen

**Affiliations:** ^1^Institute of Biological Sciences, Faculty of Science, University of Malaya, Kuala Lumpur, Malaysia; ^2^Department of Biotechnology and Biomedicine, Technical University of Denmark, Kongens Lyngby, Denmark

**Keywords:** plant biotechnology, malaria, artemisinin, *Artemisia annua*, bioengineering

## Abstract

Malaria is still an eminent threat to major parts of the world population mainly in sub-Saharan Africa. Researchers around the world continuously seek novel solutions to either eliminate or treat the disease. Artemisinin, isolated from the Chinese medicinal herb *Artemisia annua*, is the active ingredient in artemisinin-based combination therapies used to treat the disease. However, naturally artemisinin is produced in small quantities, which leads to a shortage of global supply. Due to its complex structure, it is difficult chemically synthesize. Thus to date, *A. annua* remains as the main commercial source of artemisinin. Current advances in genetic and metabolic engineering drives to more diverse approaches and developments on improving *in planta* production of artemisinin, both in *A. annua* and in other plants. In this review, we describe efforts in bioengineering to obtain a higher production of artemisinin in *A. annua* and stable heterologous *in planta* systems. The current progress and advancements provides hope for significantly improved production in plants.

## Introduction

Malaria is still a global concern with around 214 million annual cases and 430,000 annual deaths, mainly among of children younger than 5 (World Health Organization [WHO], 2016). This fatal disease is caused by *Plasmodium* sp. particularly *Plasmodium falciparum* that proliferate in female *Anopheles* mosquitoes (Cox, 2010). Since the 1940s there has been continuous attempts to halt the spread of the disease and this has succeeded in Europe, North America, and parts of Asia and Latin America (Carter and Mendis, 2002). However, not in Sub-Saharan Africa where 80% of the annual malaria patients are found. Besides measures such as vector control and insecticide-treated nets, research and development has led to new drugs and a vaccine. The current preferred therapy is artemisinin combination therapy (ACT) (Banek et al., 2014; Lalloo et al., 2016) that is based on artemisinin produced in the natural source *Artemisia annua*. Artemisinin can also be produced heterologously in the plants *Nicotiana benthamiana* and *Physcomitrella patens* (Han et al., 2016; Wang et al., 2016; Ikram et al., 2017). The vaccine toward *Plasmodium* is called PfSPZ and can be produced in *N. benthamiana* and *P. patens* plants (Rosales-Mendoza et al., 2014, 2017; Boes et al., 2016; Epstein et al., 2017).

Malaria drugs have contributed significantly to the reductions in malaria mortality and morbidity. The focus for many years has been to screen traditional medicine to find new antimalarial drugs (Simonsen et al., 2001; Adia et al., 2016; Nondo et al., 2017). The malaria drug artemisinin is an example of this and originates from *A. annua*, a Chinese medicinal plant (Qinghao), commonly known as sweet wormwood. It was discovered by the Chinese researcher You-You Tu and her team in 1972, and was named Qinghaosu (Klayman, 1985; Tu, 2011). Chemically, artemisinin is a sesquiterpene lactone with a unique endoperoxide structure, without the nitrogen containing heterocyclic ring like other antimalarial compounds (Luo and Shen, 1987). The *in planta* accumulation of artemisinin is 0.01–1.4% dry weight depending on the plant variety and artemisinin is stored in the glandular trichomes of *A. annua* (Duke et al., 1994; Van Agtmael et al., 1999; Bhakuni et al., 2001; Muangphrom et al., 2016). The current production using plants with a “low” content of artemisinin can only just cover the global need, which have led to an increase in price (Peplow, 2016). In 2006, World Health Organization (WHO) recommended artemisinin as the first-choice treatment for malaria. Rapid emergence of antimalarial drug resistance drew attention to formulation of artemisinin-based combination therapy (ACT) with artemisinin as the primary substance and is now the preferred treatment (World Health Organization [WHO], 2015).

To secure the global need of artemisinin, there are continuous and extensive efforts to enhance the production of artemisinin in the native plant *A. annua*. *A. annua* is currently the primary commercial source of artemisinin and significant breeding programs has contributed to higher artemisinin content in the plant (Ma et al., 2015; Pulice et al., 2016; Xie et al., 2016), including establishment of mutant libraries (Pandey et al., 2016). Several plant-breeding techniques have been applied to create superior cultivars of *A. annua*. For example, conventional breeding by crossing *A. annua* with high artemisinin content in wild population has led to hybrid lines with 2% artemisinin d.w (Delabays et al., 2001; Cockram et al., 2012). A detailed genetic map of *A. annua* comprising of genes and markers controlling artemisinin yield has been established to generate robust high yielding crops (Graham et al., 2010). Identification of *A. annua* superior parental lines with desired traits from these genetic maps has provided two high-yielding hybrids and diallel crossing of the parental lines and the hybrids has showed consistent results for the development of improved *A. annua* hybrids (Townsend et al., 2013). Doubling the number of chromosomes generated a new variety of tetraploid cultivar with higher artemisinin content and this might become a new elite line (Banyai et al., 2010b). The overall production of the new cultivars from various laboratories have increased the level of artemisinin to about 1 to 2% d.w. (Delabays et al., 1993; Ferreira et al., 2005; Graham et al., 2010; Brisibe et al., 2012), but not all the established plant lines are stable over generations (Delabays et al., 2001).

Efforts in plant breeding have been challenging due to the heterozygous nature of *A. annua*, which results in transgenic plants with varying degrees of artemisinin content even though they were generated in the same laboratory (Delabays et al., 2001; Graham et al., 2010; Larson et al., 2013). This variation is due to the segregation of the heterozygous wild type progeny leading to a different genetic background than the parent plant. Although several high content lines have been created, the unstable yield in the progeny of these cultivars were insufficient to increase the global supply of artemisinin (Shretta and Yadav, 2012; Paddon et al., 2013).

Accumulation of artemisinin in *A. annua* is limited to the small 10 cell glandular trichomes (GT) mostly on leaves and other aerial parts (Ferreira and Janick, 1995; Lommen et al., 2006; Ling et al., 2016). Low GT numbers are correlated to low artemisinin content (Graham et al., 2010; Kjær et al., 2012). Attempts to increase the number of GTs by physical and chemical stress have not been successful (Kjær et al., 2012). One study expressed the β glucosidase (*bgl*1) gene in *A. annua* through *Agrobacterium*-mediated transformation, which resulted in an increase of GT density by 20% on leaves and 66% on flowers and an increase in artemisinin content of 1.4% in leaves and 2.56% in flowers (d.w). Manipulating GT density together with biosynthetic pathway engineering may further increase artemisinin content in *A. annua*. In depth understanding of *A. annua* GT generation at the molecular level, will broaden the opportunities of increasing the artemisinin production. This approximately though require a greater acceptance of GMO crops in open fields to ensure the global supply.

Plant tissue culture has also been investigated to establish a production of artemisinin in *A. annua* hairy root or cell suspension cultures (Nair et al., 1986; Baldi and Dixit, 2008). Several manipulations of the growth conditions such as different sugar supply, light irradiation, UV-B radiation and chilling treatment have led to production of artemisinin in *A. annua* tissue cultures (Woerdenbag et al., 1993; Liu et al., 2002; Wang and Weathers, 2007; Baldi and Dixit, 2008; Yin et al., 2008; Pandey and Pandey-Rai, 2014). Generating somaclonal variants tolerant against salt stress through gamma-rays irradiation has resulted in 13 somaclonal variants (ASV1 to ASV13) of which one of the variants, ASV12 is a stable salt-tolerant line with a higher expression profile of artemisinin key genes (ADS, CYP71AV1, DBR2, and ALDH1) and a higher artemisinin content as compared to wild type. In addition, treatments with elicitors such as methyl jasmonate has significantly increased artemisinin production by up to 49% including up-regulating the expression of artemisinin biosynthesis genes as well as increased GT index (0.128) (Baldi and Dixit, 2008; Wang et al., 2010; Dangash et al., 2014; Xiang et al., 2015). Other elicitors such as chitosan, gibberellic acid, and salicylic acid also aid in the accumulation of artemisinin (Guo et al., 2010; Banyai et al., 2011). Combinations of various cultivation and elicitation methods are currently being geared for a mass production of artemisinin in *A. annua* hairy roots via bioreactors with 6.3 g/L dry weight (37.50 g fresh weight) biomass and 0.32 mg/g artemisinin content after 25 days (Patra and Srivastava, 2017).

Other efforts to enhance artemisinin production have been attempted through genetic engineering of the artemisinin biosynthetic pathway genes in microbial heterologous hosts. Extensive work on the development of microbial production of artemisinin precursors led to semi-synthesis of artemisinin, but this is only partly commercially successful (Benjamin et al., 2016; Peplow, 2016; Singh et al., 2017). In this review, the progress and recent bioengineering advances in artemisinin production in stable heterologous *in planta* systems including genetic modifications of *A. annua* is summarized.

## Artemisinin Biosynthesis in *Artemisia annua*

**FIGURE 1:**
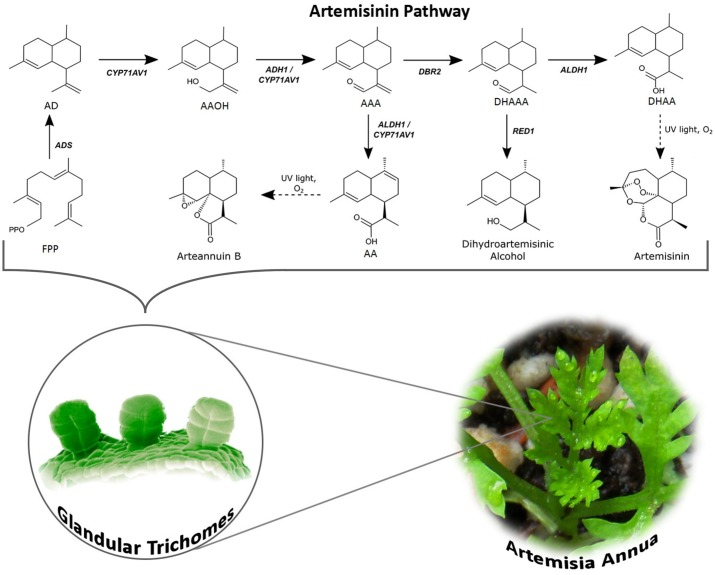
Artemisinin biosynthesis pathway occurs in the glandular trichomes of *Artemisia annua*. The pathway intermediates are defined as FPP, farnesyl diphosphate; AD, amorpha-4,11-diene; AAOH, artemisinic alcohol; AAA, artemisinic aldehyde; AA, artemisinic acid; DHAAA, dihydroartemisinic aldehyde; DHAA, dihydroartemisinic acid. The full name of the enzymes is stated in the text.

The biosynthesis of Artemisinin (**Figure 1**) has been explored for many years. However, not every detail about the regulation and biosynthesis is completely understood, but the discovery that the whole biosynthesis is located in the glandular trichomes of *A. annua* has facilitated in-depth regulatory studies (Olsson et al., 2009; Olofsson et al., 2011). Derived from the general terpenoid biosynthesis, two molecules of isopentenyl diphosphate (IPP) and one dimethylallyl diphosphate (DMAPP) are condensed by farnesyl diphosphate synthase (FPPS/FPS) into farnesyl diphosphate (FPP, farnesyl pyrophosphate), the C15 sesquiterpenoid precursor (Weathers et al., 2006; Brown, 2010; Wen and Yu, 2011). Overexpression of FPS in *A. annua* resulted in an increase of artemisinin production (Han et al., 2006; Banyai et al., 2010a), which confirms the role of FPS and availability of the substrates in the regulation of artemisinin biosynthesis similar to other sesquiterpene lactones (Simonsen et al., 2013).

FPP is converted to amorpha-4,11-diene by amorpha-4,11-diene synthase (ADS) via carbocation formation and cyclization (Bouwmeester et al., 1999; Mercke et al., 2000; Picaud et al., 2005, 2006). In the following two oxidization steps, amorpha-4,11-diene is hydroxylated into artemisinic alcohol and oxidized to artemisinic aldehyde by amorphadiene monooxygenase (CYP71AV1), a cytochrome P450 enzyme (Teoh et al., 2006; Wang et al., 2011). The activity of the CYP71AV1 has also been confirmed through a knock-out of the endogenous gene in *A. annua* showing that these plants do not produce any downstream products of amorphadiene (Czechowski et al., 2016). It has later been discovered that the alcohol dehydrogenase (ADH1, a dehydrogenase/reductase enzyme) is specific toward artemisinin alcohol and oxidizes this to the aldehyde. This specificity and strong expression in *A. annua* glandular trichomes confirms that ADH1 is responsible for oxidation of artemisinic alcohol to artemisinic aldehyde (Olofsson et al., 2011; Paddon et al., 2013; He et al., 2017). Artemisinic aldehyde is further reduced to dihydroartemisinic aldehyde by artemisinic aldehyde Δ11 (13) reductase (DBR2) and subsequently oxidized to dihydroartemisinic acid by aldehyde dehydrogenase (ALDH1), which is also expressed in the trichomes (Zhang et al., 2008; Teoh et al., 2009; Rydén et al., 2010; Liu et al., 2016). Besides catalyzing the oxidation of dihydroartemisinic aldehyde to the acid, ALDH1 also catalyzes the oxidation of artemisinic aldehyde to artemisinic acid (a reaction that in yeast is catalyzed by CYP71AV1) (Teoh et al., 2006, 2009). Another enzyme, dihydroartemisinic aldehyde reductase (RED1) converts dihydroartemisinic aldehyde to dihydroartemisinic alcohol, a “dead end” substance, which affects the production yield of artemisinin (Rydén et al., 2010). The final step is a light-induced non-enzymatic spontaneous reaction converting dihydroartemisinic acid to artemisinin and artemisinic acid to arteannuin B (Sy and Brown, 2002; Teoh et al., 2006; Czechowski et al., 2016).

## Bioengineering of Artemisinin Production in Green Plant Cells

### Bioengineering of Biosynthetic Genes in *Artemisia annua*

Characterization of enzymes in the artemisinin biosynthetic pathway provides new tools and advances the possibility of engineering the production of artemisinin. This can be achieved by enhancing the general terpenoid metabolism and through overexpression of several genes involved in artemisinin biosynthesis in *A. annua* (Tang et al., 2014). Overexpression of key terpenoid genes encoding for the enzymes IDI, FPS, HMGR, the plastid targeted DXR and HDR have increased production significantly (some by 2 to 3 fold) in many different studies in *A. annua* (Han et al., 2006; Aquil et al., 2009; Banyai et al., 2010a; Nafis et al., 2011; Xiang et al., 2012; Ma et al., 2017a). Co-expression of FPS, CYP71AV1 and its redox partner, POR (cytochrome P450 reductase) increased production by 3.6 fold, whereas combining four genes ADS, CYP71AV1, ALDH1, and POR from *A. annua* yielded a 3.4 fold increase in the artemisinin levels (Chen et al., 2013; Shi et al., 2017). Additionally, the production also increased by overexpression of ADS, CYP71AV1, and HMGR (Ma et al., 2009; Alam et al., 2016). The expression of several genes in the pathway clearly have an effect on the artemisinin level and do increase the amount of biomass obtained (Shen et al., 2012; Alam et al., 2016). Thus, utilizing genetic engineering to target the expression of both upstream and specific artemisinin genes should be pursued.

The overexpression of DBR2 clearly showed that this is a key enzyme that regulates the production of artemisinin by guiding the metabolic flow from artemisinic acid toward dihydroartemisinic acid. Without the activity of DBR2 the plants solely make artemisinic acid and thereby arteannuin B (Zhang et al., 2008), thus showing that overexpression of this enzyme will enhance artemisinin production. Collectively, the overexpression studies have provided insights into the understanding of the pathway and how to upregulate it.

Another strategy in bioengineering is to block competing reactions such as the squalene synthase (SQS) and β-caryophyllene synthase, enzymes consuming FPP for sterol and β-caryophyllene biosynthesis. This has been proven to elevate artemisinin production by 3.14 and 5.49 fold, respectively (Zhang et al., 2009; Chen et al., 2011). Since RED1 competes with ALDH1 in artemisinin biosynthesis of *A. annua*, removing RED1 could also lead to the increase of artemisinin production in *A. annua* (Rydén et al., 2010).

FPS in general has a higher *k_cat_* value than sesquiterpene synthases and this is true for the FPS and ADS in *A. annua*. Thus, it has been investigated whether a fusion of the two enzymes would increase the turnover of FPP to amorphadiene (Han et al., 2016). The findings that such fusion can facilitate metabolite channeling through a biosynthesis pathway has recently been shown for other metabolites (Laursen et al., 2016). The metabolite channeling from FPS to ADS is supported by a 2–3 fold increase of amorphadiene in plants where these two genes are fused (Han et al., 2016). The dynamic artemisinin content in the transgenic and wild type plants is associated with the expression of these genes involved in the artemisinin pathway. **Table 1** summarizes the work on genetic manipulation in *A. annua* to improve the production of artemisinin.

**Table 1:** Genetic engineering to improve the production of artemisinin in *Artemisia Annua.*

Type	Overexpressed enzymes	Artemisinin yield	Reference
Upstream key enzymes	FPS	13.0 mg/g (DW)	Han et al., 2006 Banyai et al., 2010a Chen et al., 2000
	HMGR	1.7 mg/g (DW)	Aquil et al., 2009
		0.6 mg/g (DW)	Nafis et al., 2011
	AaIPP1	2.5 mg/g (DW)	Ma et al., 2017a
	AaHDR1	0.09 mg/g (FW)	Ma et al., 2017b
Artemisinin biosynthesis enzymes	DXR	1.21 mg/g (DW)	Xiang et al., 2012
with related key enzymes	CYP71AV1 and POR	0.98 mg/g (FW)	Shen et al., 2012
	HMGR and ADS	1.73 mg/g (DW)	Alam and Abdin, 2011
	FPS and ADS	26 mg/g (DW)	Han et al., 2016
	FPS, CYP71AV1, POR	2.90 mg/g (FW)	Chen et al., 2013
	ADS, CYP71AV1, ALDH1, and POR		Shi et al., 2017
	ADS, CYP71AV1, POR	15.1 mg/g (DW)	Lu et al., 2013a
	DBR2	22.35 mg/g (DW)	Tang et al., 2012b
	ALDH1	25.34 mg/g (DW)	Tang et al., 2012a
Competitive pathway enzymes	SQS	31.0 mg/g (DW)	Zhang et al., 2009
	CPS	3.56 mg/g (DW)	Chen et al., 2011
Transcription factors	AaWRKY	14.2 mg/g (DW)	Jiang et al., 2016
	AaERF1 and AaERF2	9.1 and 8.1 mg/g (DW)	Yu et al., 2012
	AaORA	11.9 mg/g (DW)	Lu et al., 2013b
	AaMYC2	15.3 mg/g (DW)	Shen et al., 2016a
	AaNAC1	23.5 mg/g (DW)	Lv et al., 2016
Others	Rol B	7.30 ug/g (DW)	
	Rol C	3.33 ug/g (DW)	Dilshad et al., 2015b
	AaPYL9	1.80 mg/g (FW)	Zhang et al., 2013
	AtCRY1	1.65 mg/g (DW)	Hong et al., 2009

### Bioengineering the Regulation of Artemisinin Biosynthesis

Over the last 20 years *Agrobacterium rol* A, B, and C genes have been shown to increase the biosynthesis of stress response metabolites in different plant families (Bulgakov, 2008). *Rol* genes are a potential activator of secondary metabolites which directly upregulate artemisinin production by induction of the gene expression, leading to higher amounts of enzymes and thus more products. Transformation of *rol* genes in other *Artemisia* sp. resulted not only in the overexpression of artemisinin pathway genes, but also artemisinin content in the plant (Dilshad et al., 2015a,b; Amanullah et al., 2016). Integration of individual and combined *rol* B and C genes in *A. annua* increases the production of artemisinin by up to ninefold (Ghosh et al., 1997; Dilshad et al., 2015a,b).

Identifying transcription factors involved in regulating artemisinin production has also contributed to a higher production of artemisinin and was recently reviewed (Shen et al., 2016b). Overexpression of the transcription factor AaWRKY1 shows a 4.4 fold increase of artemisinin compared to the control plant. Overexpression of another transcription factor jasmonate-responsive AP2/ERF-type; AaERF1 and AaERF2 increases the gene expression levels of ADS, CYP71AV1, and DBR2 resulting in a higher accumulation of artemisinin and artemisinic acid in *A. annua* (Shen et al., 2016b). What is clear from recent work is that there are parts of the artemisinin pathway, which have promoters that are specific for trichomes (Chen et al., 2017). Therefore changing these to a strong constitutive promoter might be a novel engineering target with CRISPR/Cas9 technology.

### Metabolic Engineering in *Nicotiana* spp.

Introducing artemisinin pathway genes in heterologous plants has been successful in both stable and transient expression but the artemisinin yield is relatively low (Farhi et al., 2011; Zhang et al., 2011; Ting et al., 2013). Currently, only *Nicotiana* spp. has been used as the plant alternative in the artemisinin research as it is cheap, well-established with rapid growth and high biomass. The expression of ADS in *Nicotiana tabacum* resulted in an increased production of the first product amorpha-4,11-diene (Wallaart et al., 2001). The addition of CYP71AV1, DBR2, and ALDH1 produced 4 mg/g fresh weight of amorph-4,11-adiene in leaves followed by 0.01 mg/g dry weight of artemisinic alcohol (Zhang et al., 2011). Stable expression of five multiple genes from the MVA and artemisinin pathway constructed in a single vector into *N. tabacum* produces 0.48–6.8 μg/g dry weight of artemisinin (Farhi et al., 2011). However, transient expression combining ADS, FPS, HMGR, and CYP71AV1 in *N. benthamiana* produced artemisinic acid that was further modified by endogenous glycosyl transferase into artemisinic acid-12-β-diglucoside (Van Herpen et al., 2010). There is a high production of glycosylated artemisinin precursors with the expression of artemisinin genes in *N. benthamiana* (Ting et al., 2013).

Glycosylation is a problem in the *Nicotiana* spp. (Van Herpen et al., 2010; Ting et al., 2013). To overcome this, attempts were made to target the biosynthesis into different cellular compartments such as the chloroplast. Fuentes et al. (2016) introduced the artemisinin pathway into *N. tabacum* chloroplast via a stable plastid genome transformation followed by a combinatorial transformation resulting in a transformation of transplastomic recipient lines (COSTREL) that produces 120 μg/g artemisinic acid.

Another group aimed to engineer two mega-metabolic pathways separately into two different cellular compartments. They first elevated the IPP pools by introducing six genes from MVA pathway into *N. tabacum* chloroplast followed by the artemisinin pathway genes into the nuclear genome with subcellular targeting at DBR2, CPR, and CYP71AV1 via chloroplast transit peptide. The lines produced ∼0.8 mg/g dry weight of artemisinin (Malhotra et al., 2016). While various methods were explored in order to enhance artemisinin production in *Nicotiana*, the production levels remain minimal due to the complex nature of the gene expression and regulation in artemisinin biosynthesis pathway as well as the complex glycosylation response in *Nicotiana*.

### Metabolic Engineering in *Physcomitrella* Patens

A new production platform is being established in a non-vascular plant, the moss *P. patens* (Simonsen et al., 2009; Buttner-Mainik et al., 2011; Ikram et al., 2015; Reski et al., 2015). Having unique molecular tools of highly efficient homologous recombination and a fully sequenced genome, *P. patens* is an attractive production system when compared to other plant production hosts (Schaefer and Zrÿd, 1997; Reski, 1998; Frank et al., 2005). Additionally, a novel transformation technology involving *in vivo* assembly of multiple DNA fragments in *P. patens* has been established, further increasing its attractiveness as a promising photosynthetic chassis for synthetic biology (King et al., 2016). This is also supported by several works utilizing *P. patens* as a “green factory,” for example, the expression of taxadiene synthase in *P. patens* produces taxadiene without any phenotypic change, making it a capable host for the production of paclitaxel (Anterola et al., 2009). In addition, three important sesquiterpenoids in the fragrance industry, patchoulol, β-santalene, and sclareol was also successfully produced in *P. patens* with productivity up to 1.3, 0.039, and 2.84 mg/g dry weight respectively (Zhan et al., 2014; Pan et al., 2015). We recently reported the successful production of artemisinin in *P. patens* (Ikram et al., 2017). All five artemisinin pathway genes were introduced into *P. patens* via the *in vivo* assembly of multiple DNA fragments method and the transgenic *P. patens* lines produces 0.21 mg/g DW of artemisinin, a significant level at only 3 days of culturing. A considerable advantage of *P. patens* as an artemisinin production platform is the absence of pathway intermediates (glycosylation and glutathione conjugation). *P. patens* has less glycosyltransferases as compared to higher plants that may lead to the possibility of lower risk of endogenous modifications to xenobiotic metabolites. Further research in bioengineering of *P. patens* for a higher artemisinin production is ongoing and could potentially help stabilize the supply of artemisinin and aid in containing malaria. Bioengineering of artemisinin biosynthesis pathway in heterologous *in planta* host with successful production of artemisinic acid and artemisinin is summarized in **Table 2**.

**Table 2:** Compilation of bioengineering works in heterologous *in planta* host producing artemisinin and artemisinic acid.

Construct	Strain	Artemisinin	Artemisinic acid	Reference
Mega vector consisting HMGR, CYP71AV1, CPR, DBR2, cytosolic-targeting ADS and mitochondria-targeting ADS	*Nicotiana tabacum*	0.0068 mg/g DW	–	Farhi et al., 2011
Transient expression of ADS, CYP71AV1, DBR2, ALDH1 with co-expression of lipid transfer proteins from *A. annua*	*Nicotiana benthamiana*	0.0030 μg/g DW	–	Wang et al., 2016
FPS, ADS, CYP71AV1, CPR targeted at the chloroplast followed by combinatorial supertransformation of CYPB5, ADH1, ALDH1, DBR2	*Nicotiana tabacum*	–	120 μg/g FW	Fuentes et al., 2016
Six MVA pathway genes, AACT, HMGS, HMGR, MVK, PMK, PMD transformed into chloroplast and subcellular targeting DBR2, CYP71AV1 and CPR via chloroplast transient peptide	*Nicotiana tabacum*	0.8 mg/g DW	–	Malhotra et al., 2016
Five artemisinin pathway genes, ADS, CYP71AV1, ADH1, DBR2, ALDH1 stably transformed into *Physcomitrella patens* via a novel *in vivo* DNA assembly method	*Physcomitrella patens*	0.21 mg/g DW	–	Ikram et al., 2017

## Perspectives

Current advances in genetic and metabolic engineering drive a more diverse research and development approach on improving *in planta* production of artemisinin. The successes achieved in heterologous plant hosts and engineering of *A. annua* remains are of great importance. Microbial engineering of artemisinic acid shows some potential, but the added costs for later chemical synthesis of artemisinin is a detracting factor for replacing *A. Annua* as the main artemisinin source. Progress in plant engineering and synthetic biology has significantly improved the awareness of using plant as production hosts leading to great efforts in the implementation and enhancement of artemisinin production in both *in vivo* and *in vitro* production. Furthermore, heterologous *in planta* production seems to be more cost effective and environmentally friendly than other current biotechnological platforms. Advances in multigene transformation, transcription factors along with targeting of cellular compartment techniques will enable elevation of production levels in future engineered plants bringing us closer to industrial scale plant factories for artemisinin production. Perhaps the continuous production of artemisinin and other valuable plant metabolites in suspended bioreactor cultures with *in situ* extraction to avoid cell toxicity is not too far in the future. This will avoid the regulatory restrictions on in field GMO plants, and allow for stable continues production of drugs.

## Author Contributions

NI and HS collectively wrote the manuscript and initiated the work behind it. NI contributed with major parts of the literature research.

## Conflict of Interest Statement

The authors declare that the research was conducted in the absence of any commercial or financial relationships that could be construed as a potential conflict of interest.
